# Down-Regulated FOXO1 in Refractory/Relapse Childhood B-Cell Acute Lymphoblastic Leukemia

**DOI:** 10.3389/fonc.2020.579673

**Published:** 2020-11-11

**Authors:** Qingqing Zheng, Chuang Jiang, Haiyan Liu, Wenge Hao, Pengfei Wang, Haiying Huang, Ziping Li, Jiabi Qian, Maoxiang Qian, Hui Zhang

**Affiliations:** ^1^Department of Hematology/Oncology, Guangzhou Women and Children's Medical Center, Guangzhou, China; ^2^Shanghai Children's Medical Center, School of Medicine, Shanghai Jiao Tong University, Shanghai, China; ^3^Institute of Pediatrics and Department of Hematology and Oncology, Children's Hospital of Fudan University, the Shanghai Key Laboratory of Medical Epigenetics, International Co-laboratory of Medical Epigenetics and Metabolism, Ministry of Science and Technology, Institutes of Biomedical Sciences, Fudan University, Shanghai, China

**Keywords:** acute lymphoblast leukemia, risk stratification, relapse, *FOXO1*, MRD—minimal residual disease

## Abstract

**Background:** Acute lymphoblastic leukemia (ALL) is the most common pediatric cancer, with an overall prevalence of 4/100,000, accounting for 25–30% of all childhood cancers. With advances in childhood ALL treatment, the cure rate for childhood ALL has exceeded 80% in most countries. However, refractory/relapsed ALL remains a leading cause of treatment failure and subsequent death. Forkhead box O1 (FOXO1) belongs to the forkhead family of transcription factors, but its role in B-cell ALL (B-ALL) has not been determined yet.

**Procedures:** RNA sequencing was applied to an ALL case with induction failure (IF) to identify the possible genetic events. A cytokine-dependent growth assay in Ba/F3 cells was used to test the leukemic transformation capacity of MEIS1–FOXO1. The propidium iodide (PI) staining method was used to evaluate the effect of MEIS1–FOXO1 on cycle distribution. FOXO1 transactivity was examined using a luciferase reporter assay. *FOXO1* mRNA expression levels were examined using real-time quantitative PCR among 40 children with B-ALL treated with the CCCG-ALL-2015 protocol. Association analysis was performed to test the correlation of *FOXO1* transcription with childhood B-ALL prognosis and relapse in a series of GEO datasets. An MTT assay was performed to test the drug sensitivity.

**Results:** In this ALL case with IF, we identified a novel *MEIS1*–*FOXO1* fusion gene. The transactivity of MEIS1–FOXO1 was significantly lower than that of wild-type FOXO1. *MEIS1*–*FOXO1* potentiated leukemia transformation and promoted Ba/F3 cell cycle S-phase entry. Low *FOXO1* transcription levels were found to be strongly associated with unfavorable ALL subtype, minimal residual disease (MRD) positivity, and relapse. Lower *FOXO1* expression was associated with prednisone and cyclophosphamide resistance.

**Conclusions:** Low *FOXO1* transcription was associated with high-risk stratification and relapse in children with B-ALL, probably due to multi-drug resistance.

## Introduction

Acute lymphoblastic leukemia (ALL) is the most common pediatric cancer, with an overall prevalence of 4/100,000, accounting for 25–30% of all childhood cancers (1). The cure rate for childhood ALL has exceeded 80% in most countries and even higher than 90% in developed countries with contemporary therapy (2). However, refractory/relapsed ALL (R/R-ALL) remains to be a leading cause of treatment failure and subsequent death (3). Although most patients can achieve quick and long-term responses to contemporary chemotherapy, a non-ignorable portion of childhood ALL patients do not respond well or relapse during chemotherapy. Cumulating evidence has pointed out that the long-term outcome of patients with relapsed or induction failure (IF) is very dismal. Thus, precise risk stratification at an early stage is very essential for directing patients into more optimized therapy regimens.

Importantly, genomic lesions play a deterministic role in R/R-ALL. For example, patients with Philadelphia chromosome (Ph) translocation, *PDGFRB*-rearrangement, *MEF2D*-rearrangement, *KMT2A*-rearrangement, *TP53* mutation, and *TCF3*-*HLF* are classified into high- or very high-risk ALL subgroups (4). Meanwhile, the gene expression profile (e.g., Ph-like signature) can predict the therapeutic response and relapse of ALL (5). Many findings have been translated into drug discovery, which improved clinical application. An example of the result of such finding is the milestone BCR-ABL1 targeted tyrosine kinase inhibitor, imatinib (6). However, a considerable portion of clinical failure cannot be entirely explained by our current knowledge. Thus, studies on ALL biology and refractoriness/relapse prediction are necessary for the early identification of new driver alterations and subsequent treatment with more aggressive strategies, such as chimeric antigen receptor (CAR) T-cell therapy or hematopoietic stem cell transplantation (HSCT) (7).

In this study, we identified a novel forkhead box O1 (*FOXO1*) fusion gene, namely, *MEIS1*–*FOXO1*, in a B-cell ALL (B-ALL) case with IF. Using the Ba/F3 transformation model, we found that *MEIS1*–*FOXO1* could potentiate leukemogenesis *in vitro* and cell cycle S-phase entry. Furthermore, the transcription activity of the MEIS1–FOXO1 fusion protein was completely abolished as compared with its wild-type FOXO1 protein. Gene expression correlation analysis identified that lower *FOXO1* transcription levels were associated with high-risk stratification and relapse in children with B-ALL. Finally, we tested the role of FOXO1 in drug response and found that lower *FOXO1* expression was associated with prednisone and cyclophosphamide resistance.

## Patients and Methods

### Patients

The patients were prospectively enrolled in the CCCG-2015-ALL clinical trial, which was approved by the institutional review board of the Guangzhou Women and Children Medical Center (GWCMC) (2018022205). Details of the enrollment criteria and study design have been described previously (8). All the investigated pediatric ALL patients were treated in the GWCMC. This study was approved by the Institutional Ethics Committee of the GWCMC (IRB nos. 2018022205, 2017102307, 2015020936, and 2019-04700), registered at the Chinese Clinical Trial Registry (ChiCTR-IPR-14005706), and conducted in accordance with the Declaration of Helsinki. Informed consent was obtained from the patients or their legal guardians.

### Next Generation Sequencing and Validation

TruSeq stranded mRNA library prep kit (Illumina) was used for whole-transcriptome library preparation, and paired-end sequencing was performed using the Illumina HiSeq 2,000/2,500 platform with a 101-bp read length at Berry Genomics, Beijing. Panel sequencing of hematological malignancy-related genes (Supplementary Table 1) was performed at Kindstar Global (Beijing) Technology, Inc. Sequencing reads were aligned to the human genome (hg19) reference sequence using TopHat2 (v2.0.12) (9). *MEIS1*–*FOXO1* fusion was validated by PCR amplification of breakpoint region of the chimeric transcript in this patient's cDNA using primers listed in Supplementary Table 2, followed by Sanger sequencing.

### Cytokine-Dependent Growth Assay in Ba/F3 Cells

Full-length *FOXO1, MEIS1*, and *MEIS1*–*FOXO1* were amplified and cloned into the cL20c-IRES-GFP lentiviral vector. Lentiviral supernatants were produced by transient transfection of HEK-293T cells using calcium phosphate. The MSCV-JAK2^R683G^-IRES-GFP construct was a gift from Dr. Jun Yang at St. Jude Children's Research Hospital (10). It was modified into MSCV-JAK2^R683G^-IRES-mCherry, and retroviral particles were produced using 293T cells. Ba/F3 cells were maintained in a medium supplemented with 10 ng/ml recombinant mouse interleukin 3 (IL-3) (PeproTech). Ba/F3 cells were transduced with lentiviral supernatants expressing *FOXO1, MEIS1*, or *MEIS1*–*FOXO1*. GFP-positive cells were sorted 48 h after lentiviral transduction and maintained in an IL-3 medium for another 24 h before transfection with *JAK2*^*R*683*G*^ retroviral supernatants. Forty-eight hours later, GFP/mCherry double-positive cells were sorted and maintained in a medium with respective cytokines for 48 h. Then, the cells were washed three times and grown in the absence of cytokines. Cell viability was monitored daily with Trypan blue using a TC10 automated cell counter (BIO-RAD). Each experiment was performed in triplicates.

### Luciferase Reporter Assays

The full-length *FOXO1, MEIS1*, and *MEIS1*–*FOXO1* were amplified and cloned into the cL20c-IRES-GFP lentiviral vector and used for luciferase reporter assays to test their transactivation capability on the genes with conserved FOXO1 binding sites in HEK-293T cells. Lentiviral vectors expressing *MEIS1*–*FOXO1* and pGL.3 reporter constructs containing FOXO1 binding sites were co-transfected into HEK-293T cells. Cells were lysed 24 h after transfection with passive lysis buffer (Promega, E1910). Luciferase activity was measured using a dual-luciferase reporter assay on a Lumat LB9507 luminometer. Experiments were performed in triplicates. To control for cell number and transfection efficiency, firefly luciferase activity was normalized to Renilla luciferase.

### Quantitative Real-Time Polymerase Chain Reaction

Total RNA was extracted using the RNeasy Micro kit (Qiagen) according to the manufacturer's protocol. Five hundred nanograms of total RNA from patient samples (Supplementary Tables 3, 4) was reverse transcribed into cDNA, and real-time quantitative PCR (qRT-PCR) was performed using an ABI Prism 7900HT detection system (Applied Biosystems) with FastStart SYBR Green Master mix (Roche). *GAPDH* was used as an internal control. Primers used were listed in Supplementary Table 2.

### Gene Expression Analysis

The raw gene expression data and clinical data from four cohorts of childhood ALL patients were provided by other research groups (11–15). The relative gene expression levels (fragments per kilobase of transcript per million mapped reads, FPKM) were estimated based on supporting reads retrieved from the datasets. The FPKM values were log_2_ transformed for subsequent analyses and plotting. A two-sided *t*-test was used to validate the significance of the observed differences.

### *In vitro* Cytotoxicity Assay

Cells were seeded in 96-well plates at 25,000 cells per 100 μl per well with either vehicle (DMSO 0.1%) or increasing concentrations of drugs for 72 h. Cell viability was assessed by adding MTT reagent (Sigma) according to the manufacturer's instructions. Procedures to determine the effects of certain conditions on cell proliferation were performed in three independent experiments.

### Statistical Analysis

All statistical analyses were performed using GraphPad Prism® and/or R (version 3.2.5, https://www.R-project.org); all tests were two-sided. *P* < 0.05 was considered to be statistically significant, ^*^P < 0.05, ^**^P < 0.01, ^***^P < 0.001, and ^****^P < 0.0001.

## Results

### Identification of a Novel *MEIS1*–*FOXO1* Fusion Gene in a B-ALL Case With Induction Failure

**Figure 1:**
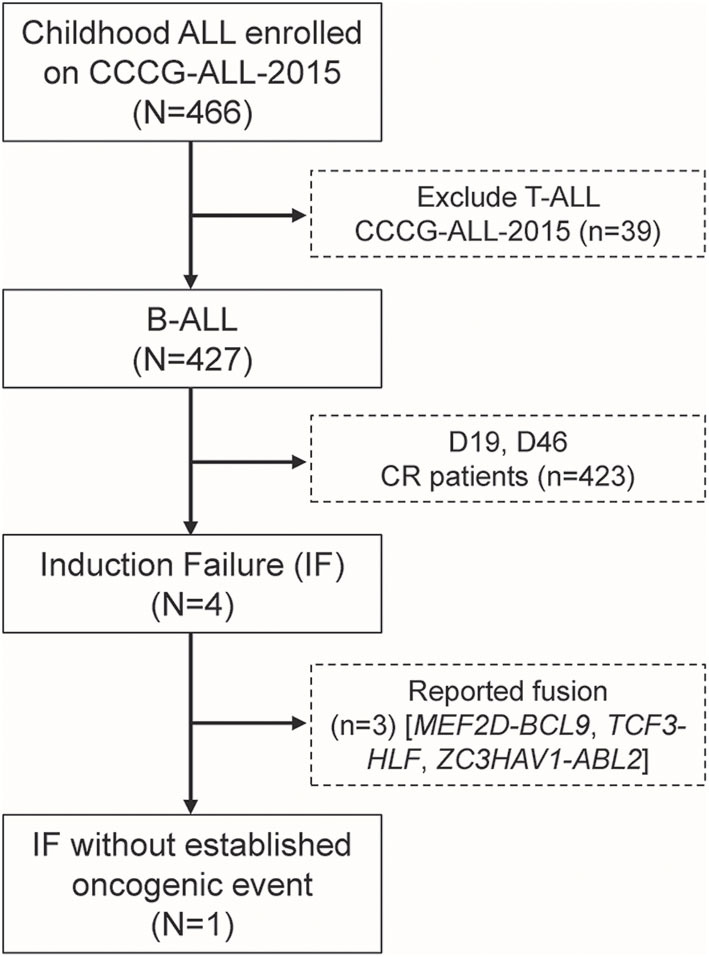
Identification of one induction failure B-ALL case without known fusion gene among patients enrolled onto CCCG-ALL-2015 in Guangzhou Women and Children's Medical Center.

**Figure 2:**
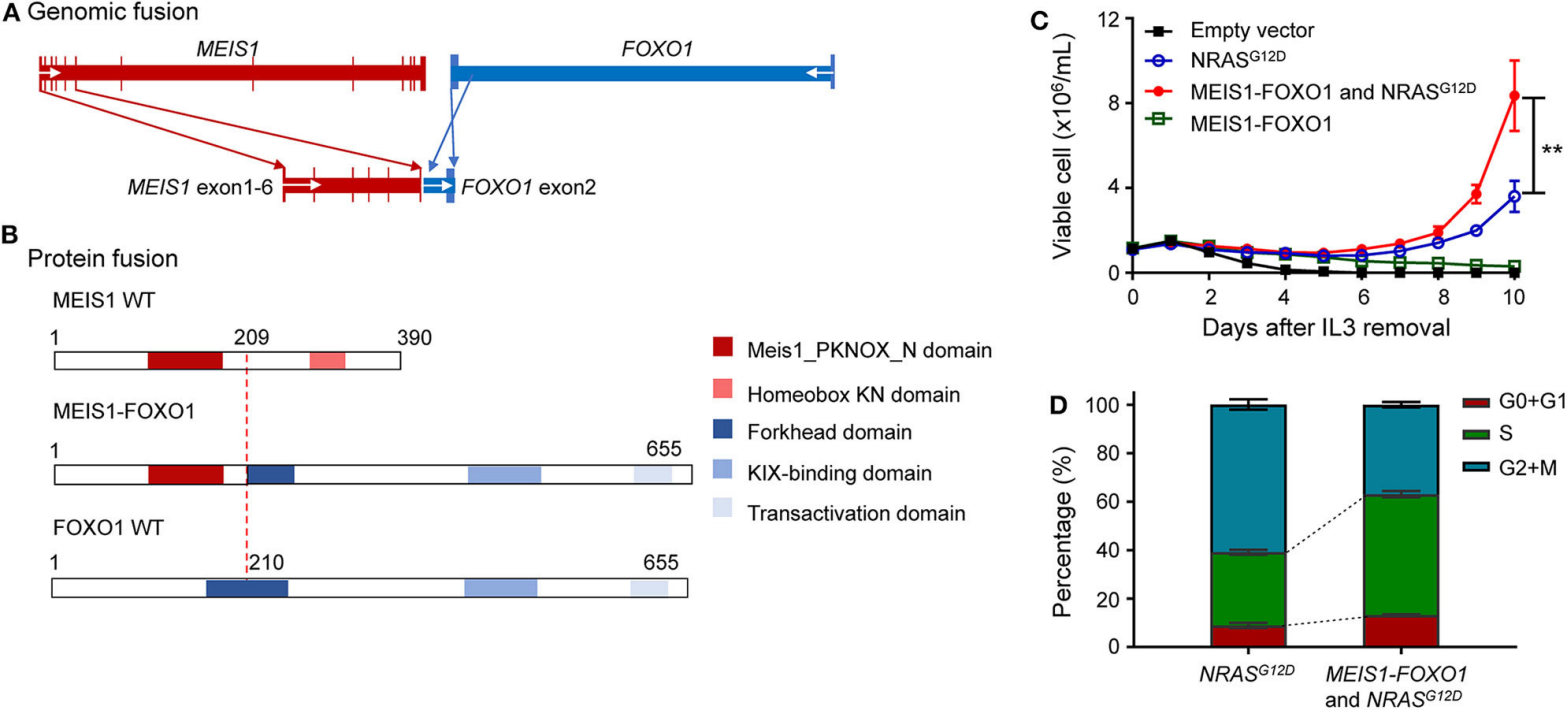
Identification of oncogenic potential of *MEIS1–FOXO1* fusion gene. **(A)** Schematic DNA representation of novel *MEIS1–FOXO1* rearrangement (red, *MEIS1*; blue, *FOXO1*). **(B)** Fusion protein representation of novel *MEIS1–FOXO1* rearrangement (red box, MEIS1 PKNOX N domain; pink box, homeobox KN domain; dark blue box, forkhead domain; light blue box, KIX-binding domain; gray blue box, transactivation domain). **(C)** MEIS1–FOXO1 potentiated leukemia transformation in Ba/F3 cell model. Effects of *MEIS1*–*FOXO1* fusion genes on Ba/F3 transformation. Following transduction of empty vector, *MEIS1*–*FOXO1, NRAS*^*G*12*D*^, or combination, Ba/F3 cells were cultured in IL-3 depleted medium with cytokine-independent cell growth as a measure of oncogenic transformation. Number of viable cells was evaluated daily. **(D)** MEIS1–FOXO1 fusion genes and proliferation of mouse hematopoietic progenitor cell Ba/F3. Ba/F3 cells were lentivirally transduced with empty vector (black), *NRAS*^*G*12*D*^ (blue), *MEIS1–FOXO1* (green), or combination of *NRAS*^*G*12*D*^ and *MEIS1–FOXO1* (red) and then cultured in the presence of IL-3 (10 ng/ml). After 48 h, cell cycle distribution was evaluated using standard PI staining protocol. Statistical significance, determined using the two-sided unpaired *t*-test, is indicated by **P < 0.01.

A total of 466 children with ALL were enrolled from March 2015 to June 2020 in the CCCG-ALL-2015 study of Guangzhou Women and Children's Medical Center, of which 427 were children with B-ALL. Around 0.94% (4 out of 427) of enrolled B-ALL patients did not respond to induction remission therapy and were classified as IF ALL (median age, 8.6 years; range, 2.1–11.9 years) (Figure 1), which was consistent with reports from other study groups (16). Among these, three IF cases were found to have known fusion genes (i.e., *MEF2D*-*BCL9, TCF3*-*HLF*, and *ZC3HAV1-ABL2*, respectively). The *MEF2D*-*BCL9* and *TCF3*-*HLF* fusion genes are well-established and classified into poor prognostic ALL subtypes (17–19), and *ZC3HAV1-ABL2* was designated as Ph-like subtype (5). Interestingly, this 2.1-year-old precursor B-ALL boy with IF could not be explained by known molecular events contributing to this treatment response (Supplementary Figure 1). At the end of induction remission therapy, 20.5% of lymphoblastic cells were detected in the bone marrow smear samples, and the minimal residual disease (MRD) level detected by flow cytometry was 10.3% (Supplementary Figure 1A). Regular pathology tests showed that he was a B-cell precursor ALL (BCP-ALL) patient with abnormal 46,XY,del(17)(p11)[15]/46,XY,i(17)(q10)[2]/46,XY[3] karyotype. Using a capture sequencing, we also identified the *NRAS*^*G*12*D*^, *TP53*^*R*273*H*^, *ABCC1*^*R*1176*X*^, *PHGR1*^*H*37*P*^, *HOXA3*^*P*219*L*^, and *DST*^*P*4606*L*^ mutations. No fusion gene was identified using the current panel RT-PCR assay (Supplementary Figures 1C–E). To determine the possible cause of IF, we performed RNA sequencing and found a novel *MEIS1–FOXO1* fusion gene that was an in-frame fusion of exon 1–6 of *MEIS1* with exon 2 of the *FOXO1* gene, which was confirmed by RT-PCR and Sanger sequencing (Figures 2A,B and Supplementary Figures 2A,B).

### The Oncogenic Potential of *MEIS1*–*FOXO1*

These findings prompted us to ask whether this novel *MEIS1*–*FOXO1* fusion gene drives B-ALL leukemogenesis and contributes to poor treatment response. To address this question, we used an IL-3-dependent growth mouse hematopoietic progenitor cell line Ba/F3 as a study model and tested whether *MEIS1–FOXO1* had some kind of oncogenic potential. Since somatic *NRAS* mutations have been reported to be sufficient for transforming leukemogenesis, we used *NRAS*^*G*12*D*^ as our experimental control. Consistent with the reports by Shannon and Castilla (20, 21), our *in vitro* assay showed that ectopic NRAS^G12D^ expression potentiated Ba/F3 cells IL-3-independent growth (Figure 2C). Although MEIS1–FOXO1 was not sufficient to transform Ba/F3 cells into IL-3-independent growth, it indeed potentiated the survival of Ba/F3 cells compared with cells transfected with mock vector (Figure 2C and Supplementary Figure 3). Furthermore, the combination of MEIS1–FOXO1 and NRAS^G12D^ accelerated Ba/F3 cells into IL-3-independent growth as compared with NRAS^G12D^ alone (Figure 2C). Using the same cell model, we tested the impact of MEIS1–FOXO1 on cell cycle distribution and found that the cotransduction of MEIS1–FOXO1 and NRAS^G12D^ potentiated S-phase entry in comparison with NRAS^G12D^ alone (Figure 2D). These results suggest the oncogenic potential of *MEIS1*–*FOXO1*.

### Lower *FOXO1* Transcription in This Induction Failure B-ALL Case

**Figure 3:**
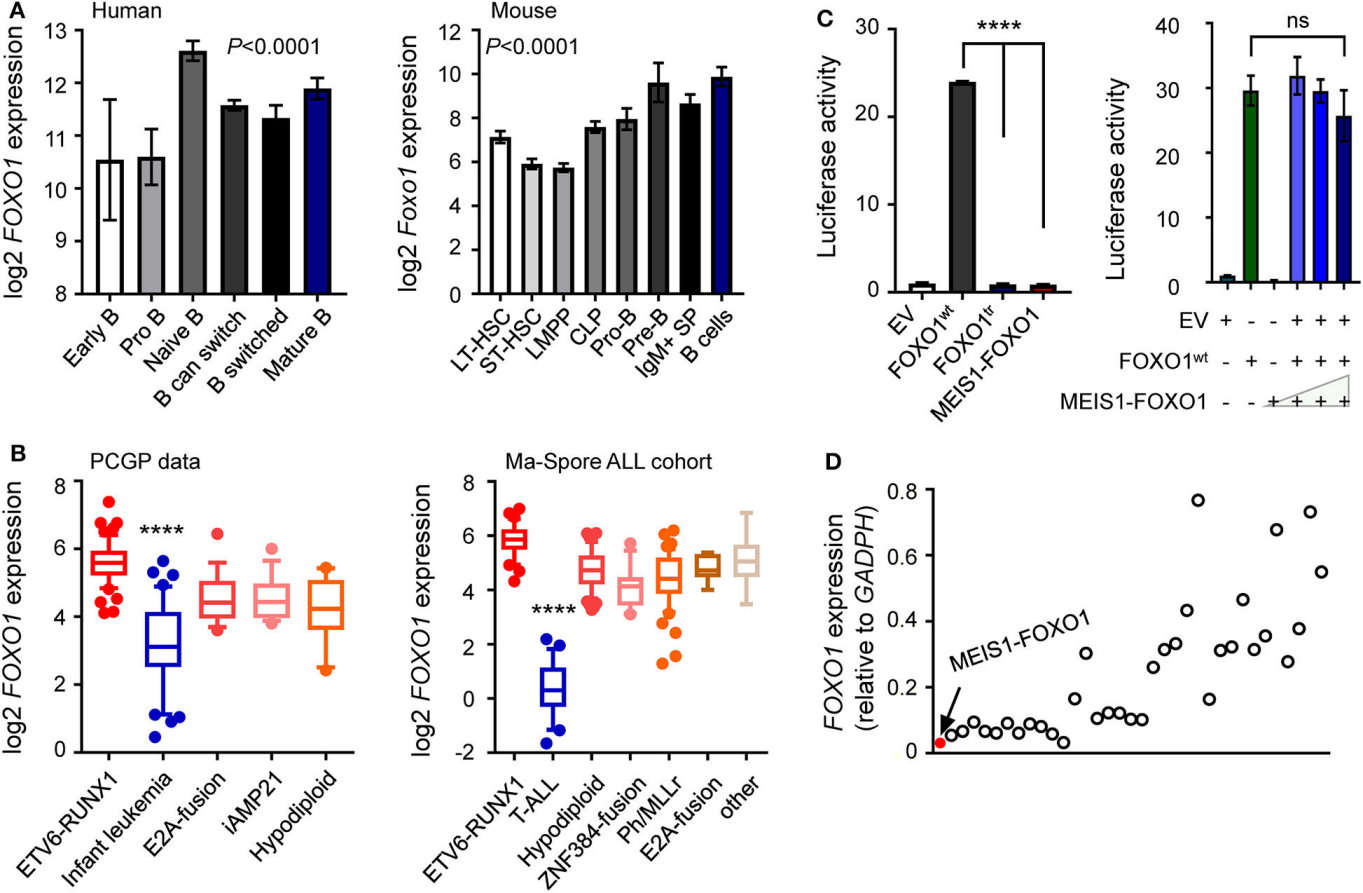
Lower *FOXO1* transcription associated with poor prognosis. **(A)** Expression of the *FOXO1* was increased along with human (left panel) and mouse (right panel) B lymphocyte development. Each B-cell group is represented by a bar and is color-coded according to the subgroups it belongs to. **(B)** Expression of the *FOXO1* was constitutively activated among B-ALL samples, with the highest expression in *ETV6*-*RUNX1* subtype. **(C)** Luciferase reporter gene assay of MEIS1–FOXO1 transcription activity. HEK-293T cells were transiently transfected with pGL3 construct (luciferase gene with FOXO1 binding sites), pcDNA construct [empty vector, wild-type FOXO1 [FOXO1^wt^], MEIS1–FOXO1, or truncated FOXO1 [FOXO1^tr^]], and pGL-TK (Renilla luciferase). **(D)** Expression of the *FOXO1* among 35 B-ALL cases. RT-PCR was performed to quantify the *FOXO1* transcription, and the quantification was expressed as relative to internal *GAPDH* control. The red dot represented the B-ALL case with *MEIS1*–*FOXO1* fusion gene. Statistical significance, determined using one-way ANOVA test **(A,B)** or two-sided unpaired *t*-test **(C)**, is indicated by ****P < 0.0001.

Accumulating evidence has demonstrated that *FOXO1* is a crucial regulator of B-cell development, in which FOXO1 inactivation causes differentiation blockage at the pro-B-cell stage (22–25). To investigate the role of *MEIS1*–*FOXO1* in B-ALL, we first examined the gene expression of fusion partners in normal hematopoiesis and B-ALL patient samples. As shown in Figure 3A, a gradual up-regulation of *FOXO1* expression was observed during B-cell differentiation, whereas *MEIS1* was downregulated, suggesting an important role of *FOXO1* in B-cell development (Figure 3A and Supplementary Figure 4A). In B-ALL samples, we found that *FOXO1* was constitutively expressed in B-ALL cells, whereas *MEIS1* was merely expressed, again suggesting the role of *FOXO1* in B-ALL (Figure 3B and Supplementary Figures 4B,C). To test the biological functions of *MEIS1*–*FOXO1*, we first utilized a luciferase reporter assay to determine the impact of MEIS1–FOXO1 on the transactivity. As shown in Figure 3C and Supplementary Figure 5, FOXO1 transactivity was abolished entirely in MEIS1–FOXO1 as compared with wild-type FOXO1. We then tested whether MEIS1–FOXO1 had a dominant-negative effect on its wild-type FOXO1 protein. As shown in Figure 3C, no dominant-negative effect was observed. Furthermore, we quantified the *FOXO1* gene expression using RT-qPCR and found that *FOXO1* was nearly not expressed in the leukemic cells of this patient with MEIS1–FOXO1 fusion (Figure 3D). Together, low *FOXO1* transcription might have contributed to IF in this patient.

### Lower *FOXO1* Transcription Might Be Associated With Poor Outcomes in Children With B-ALL *via* Drug Resistance

**Figure 4:**
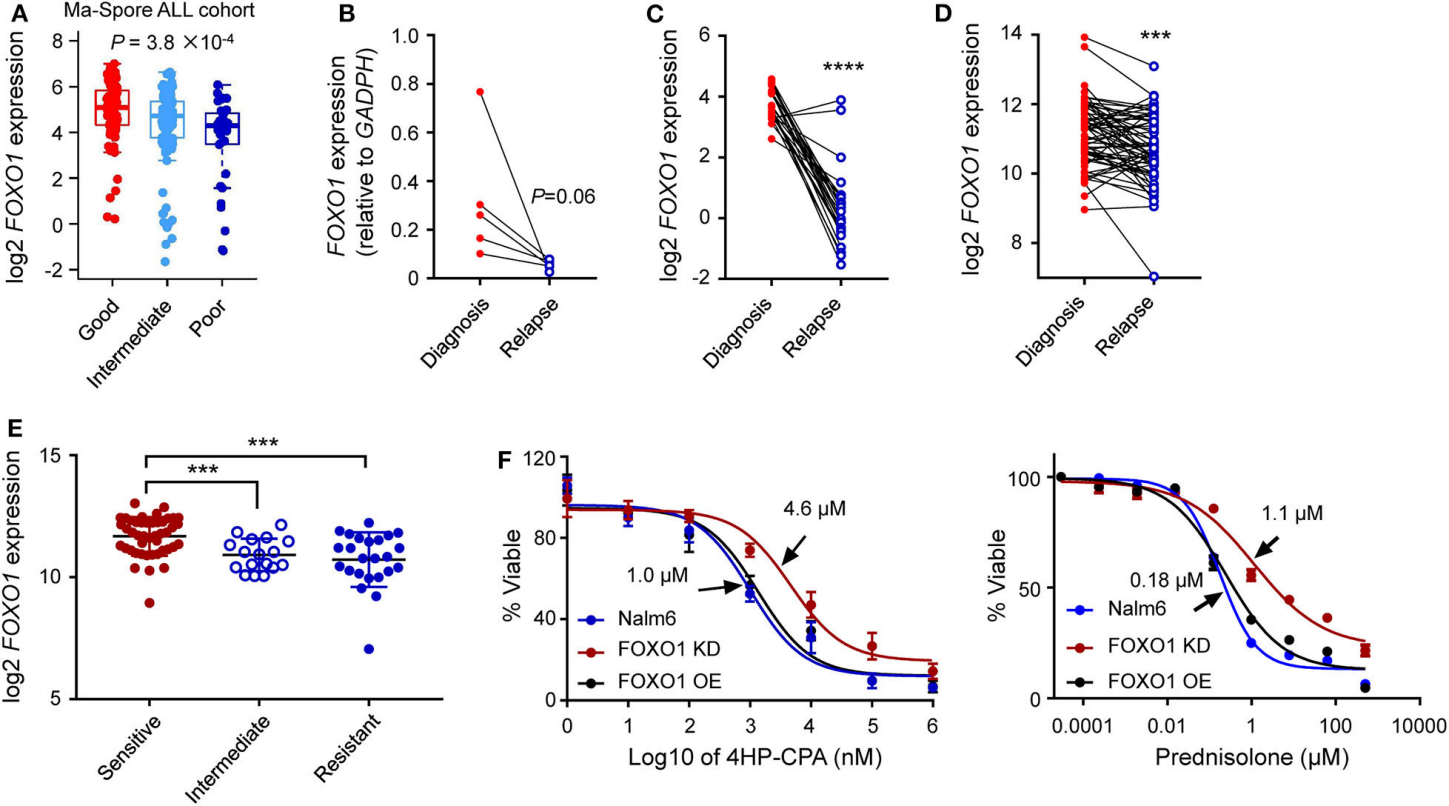
Lower *FOXO1* transcription correlated with ALL relapse. **(A)** Lower *FOXO1* was associated with MRD positivity at the end of induction. MRD at the end of induction therapy Day 33 was used: patients with MRD <0.01% or >1% were classified as “good” or “poor,” respectively, and others were classified as “intermediate.” **(B,C)** Lower *FOXO1* expression was found in relapsed B-ALL as compared with primary samples in our institutional data **(B)** and PCGP data **(C)**. **(D)** The FOXO1 transcription was extremely low in relapsed B-ALL among the diagnosis-relapse matched paired samples. **(E)** Lower FOXO1 expression was associated with glucocorticosteroids resistance. **(F)** Cytotoxicity of prednisone and 4HP-CPA was examined in Nalm6 cells with FOXO1 knockdown (dark red line, FOXO1 KD), over-expression (black line, FOXO1 OE), or parental cells (blue line). Cells were incubated with drugs for 72 h, and viability was then measured using MTT assay. Experiments were performed in triplicate and repeated at least three times. Statistical significance, determined using two-sided unpaired *t*-test **(A,E)** or two-sided paired *t*-test **(B–D)**, is indicated by ***P < 0.001 and ****P < 0.0001.

These findings prompted us to ask whether *FOXO1* expression was associated with the prognosis of B-ALL. To test our hypothesis, we retrieved and analyzed the *FOXO1* expression data from the Pediatric Cancer Genome Project (PCGP) (26) and found that *FOXO1* gene expression was the highest in *ETV6*-*RUNX1* ALL and lowest in infantile leukemia. Notably, *FOXO1* expression was significantly lower in patients with intermediate or high-risk ALL than in *ETV6*-*RUNX1* ALL, a well-known excellent prognosis group (Figure 3B and Supplementary Figure 6). Next, we applied the same strategy to analyze the data from the Ma-Spore ALL cohort (27) and observed the same pattern (Figure 3B). Moreover, in the Ma-Spore ALL cohort, we found that lower *FOXO1* expression was associated with higher MRD burden post-induction remission therapy (*P* = 3.8 × 10^−4^ by Wilcoxon rank-sum test, Figure 4A).

To evaluate the impact of *FOXO1* on B-ALL relapse, we examined the *FOXO1* mRNA levels in diagnostic and relapsed B-ALL samples from our single institution using RT-qPCR assay. As shown in Supplementary Figure 7A, *FOXO1* transcription was significantly higher in the diagnosed ALL samples than in the relapsed samples, suggesting that lower *FOXO1* transcription might be an essential index for B-ALL relapse. This expression pattern was also observed in the St. Jude PCGP dataset (Supplementary Figure 7B). To further validate this finding, we next tested the *FOXO1* transcription among five paired diagnosis-relapse samples in our study cohort and identified an extremely low *FOXO1* expression in the relapsed samples as compared with their diagnostic counterparts (Figure 4B). This observation was validated in the PCGP and the matched diagnosis-relapse dataset created by Hogan et al. (13) (Figures 4C,D), consolidating the role of *FOXO1* in B-ALL relapse. To preliminarily explore how lower *FOXO1* expression is linked with higher MRD levels and relapse, we performed a drug resistance association analysis on the datasets published by Paugh et al. (28). Of note, lower *FOXO1* expression significantly correlated with glucocorticoid resistance (Figure 4E), a key component in ALL therapy. Next, we knocked down *FOXO1* expression in Nalm6, a B-ALL leukemia cell line, and then examined the drug response. As shown in Figure 4F, Nalm6 cells with lower *FOXO1* transcription were relatively resistant to prednisone (IC50 = 1.1 μM in *FOXO1* knockdown and 0.18 μM in Nalm6 cells) and cyclophosphamide (IC50 = 4.6 μM in *FOXO1* knockdown and 1.0 μM in Nalm6 cells). Using the Ba/F3 cell model, we tested the role of *MEIS1–FOXO1* in drug response. Consistent with the role of FOXO1, the introduction of *MEIS1–FOXO1* also induced cyclophosphamide resistance in Ba/F3 cells transformed by *NRAS*^*G*12*D*^. However, the impact on prednisone resistance was very moderate (Supplementary Figure 8).

## Discussion

R/R-ALL is the priority issue for clinicians and translational researchers. Multiple layers of influencing factors, that is, specific somatic genomic lesions, inherited variation, micro-environment, and acquired mutations, play essential roles in R/R-ALL. In this study, we identified a novel *FOXO1* fusion gene in an IF B-ALL patient, namely, *MEIS1*–*FOXO1*. The frequency of MEIS1–FOXO1 in our study cohort is 0.23%. In the PCGP dataset, we have found one case (0.17%) with MEIS1–FOXO1 fusion out of 565 B-ALL cases. Except for the novel fusion gene, we have identified several gene mutations (i.e., *ABCC1*^*R*1166*X*^, *HOXA3*^*P*219*L*^, *DST*^*P*4606*L*^, *NRAS*^*G*12*D*^, and *TP53*^*R*273*H*^) (Supplementary Figures 1, 2), among which, the role of *ABCC1*^*R*1166*X*^, *HOXA3*^*P*219*L*^, and *DST*^*P*4606*L*^ is less known in the context of childhood ALL. Regarding *NRAS*^*G*12*D*^ and *TP53*^*R*273*H*^ mutations, Irving et al. (29) have reported that *NRAS* and *TP53* mutations were associated with an increased risk of progression.

As a novel fusion gene*, MEIS1*–*FOXO1* has oncogenic potential, as evidenced by the fact that it prolonged Ba/F3 survival independent of IL-3 when transduced alone, accelerated Ba/F3 cell leukemic transformation, and potentiated cell S-phase entry when co-transduced with *NRAS*^*G*12*D*^ as compared with transduction of *NRAS*^*G*12*D*^ alone. We also noticed that the MEIS1–FOXO1 protein did not negatively impair wild-type FOXO1 protein function, and *FOXO1* was nearly not expressed in this patient, indicating that low FOXO1 expression might be the cause of IF. However, the exact molecular impact of MEIS1–FOXO1 in B-ALL leukemogenesis and development needs to be explored in the future.

Recent studies have shown that FOXO1 is a predominant transcription factor in B-lineage-restricted progenitor cells. Pre-BCR signaling activation can suppress FOXO1 transcription activity and subsequent B-ALL cell maintenance (30). The novel MEIS1–FOXO1 fusion protein was deficient in binding FOXO1-regulated genes but did not affect wild-type FOXO1 protein in a dominant-negative fashion *in vitro*, suggesting that the wild-type allele can be functional. Interestingly, we noticed that the FOXO1 expression was deficient in this patient with MEIS1–FOXO1 fusion, suggesting an underlying mechanism *in vivo* mediating the low expression of the wild-type allele of *FOXO1*. Notably, we also found that there was a correlation between low FOXO1 transcription and ALL relapse among the enrolled patients and subjects in public datasets, and FOXO1 transcription was almost silenced in those relapsed patients as compared with their diagnostic counterparts, suggesting that FOXO1 status can be a valuable prognostic feature in ALL. Of note, our results showed that the FOXO1 transactivity was almost completely abolished in the MEIS1–FOXO1 protein, whereas its MEIS1 transactivity seemed regular, suggesting that MEIS1 may not play an essential role in ALL pathogenesis in this context.

*FOXO1* belongs to the forkhead family of transcription factors, which play roles in myogenic growth and differentiation, cancer development, and therapy (31–34). Fusions of *FOXO1* have been found in pediatric alveolar rhabdomyosarcoma and childhood B-ALL (35). Interestingly, while two BCP-ALL cases with *FOXO1* fusion have been reported, the exact fusion partner and the role of *FOXO1* in B-ALL remain unclarified (36). To address this question, we identified a novel *MEIS1–FOXO1* fusion by RNA-seq and examined the association of FOXO1 expression with risk stratification in multiple datasets. We found that the FOXO1 was highly expressed in ALL patients with ETV6-RUNX1 fusion (a well-known excellent prognosis group) and significantly low expression in ALL subtypes with known poor prognosis (e.g., infantile leukemia, KMT2A-rearranged, and T-ALL), and this pattern was confirmed in different cohorts (33). We also observed a correlation between lower FOXO1 expression and higher MRD burden post-induction therapy (*P* = 3.8 × 10^−4^). Intriguingly, we found that lower FOXO1 expression was significantly associated with glucocorticoid resistance, a crucial component in the ALL therapy, which may explain how lower FOXO1 expression contributes to higher MRD levels and relapse.

In conclusion, we identified a novel fusion gene of *MEIS1*–*FOXO1* and first reported the association of reduced *FOXO1* expression with ALL high-risk stratification and relapse. Our findings suggest that FOXO1 status may be a predictive marker for B-ALL risk stratification and relapse.

## Data Availability Statement

Publicly available datasets were analyzed in this study. This data can be found at: https://www.ncbi.nlm.nih.gov/geo.

## Ethics Statement

The studies involving human participants were reviewed and approved by Institutional Ethics Committee of Guangzhou Women and Children's Medical Center. Written informed consent to participate in this study was provided by the participants' legal guardian/next of kin.

## Author Contributions

HZ, CJ, HL, MQ, and QZ designed the research, analyzed the results, and wrote the paper. HZ, CJ, ZL, and JQ performed the experiments. HZ, PW, WH, HH, and QZ recruited and followed up the patient. All authors contributed to the article and approved the submitted version.

## Conflict of Interest

The authors declare that the research was conducted in the absence of any commercial or financial relationships that could be construed as a potential conflict of interest.
