# Supplementary material for: Down-Regulated FOXO1 in Refractory/Relapse Childhood B-Cell Acute Lymphoblastic Leukemia
Source: Front Oncol. 2020 Nov 11;10:579673. doi: 10.3389/fonc.2020.579673 (PMC7686545; doi:10.3389/fonc.2020.579673)
Supplement: Supplementary file 1 [file Data_Sheet_1.pdf]

Supplementary Figure 1

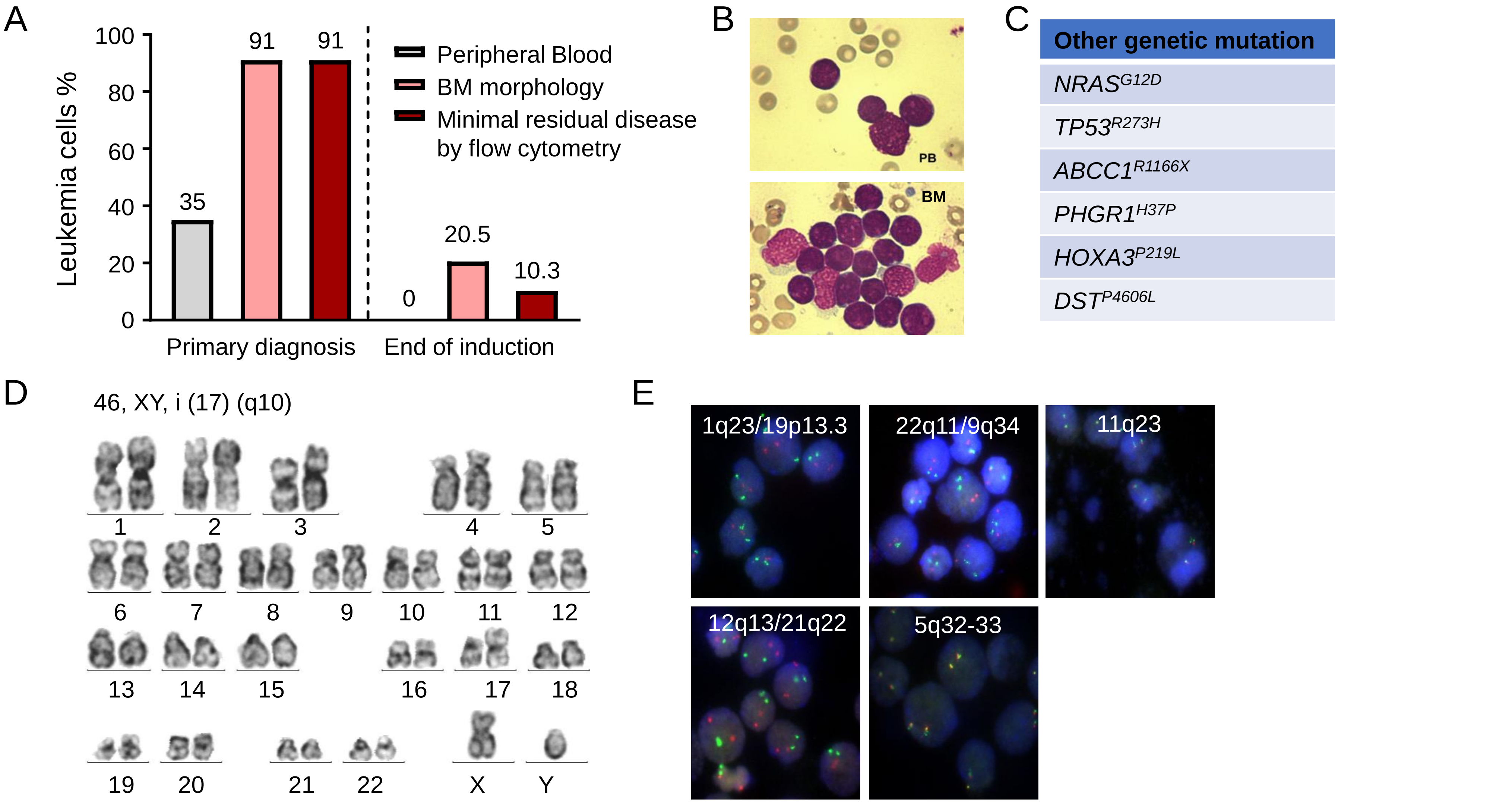

Supplementary Figure 1. MICM feature of induction failure B-ALL case. (**A**) Treatment response, (**B**) morphology, (**C-E**) cytogenetics, karyotype and fluorescence in situ hybridization test in this induction failure B-ALL case.

Supplementary Figure 2

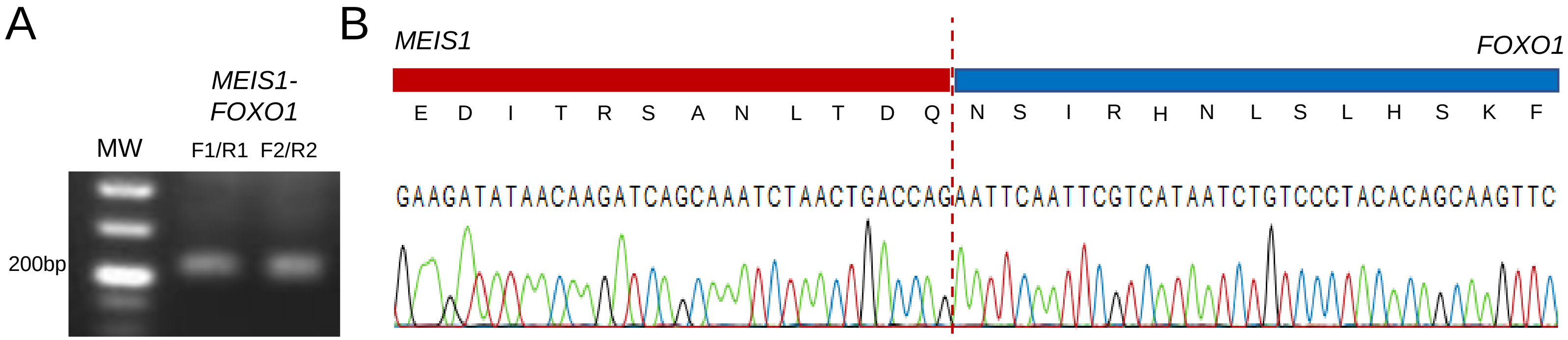

Supplementary Figure 2. Validation of novel *MEIS1-FOXO1* fusion in this induction failure case. (**A**) Reverse transcription-Polymerase Chain Reaction (RT-PCR) was performed to validate the existence of *MEIS1-FOXO1*. (**B**) Sanger sequencing of *MEIS1-FOXO1*.

Supplementary Figure 3

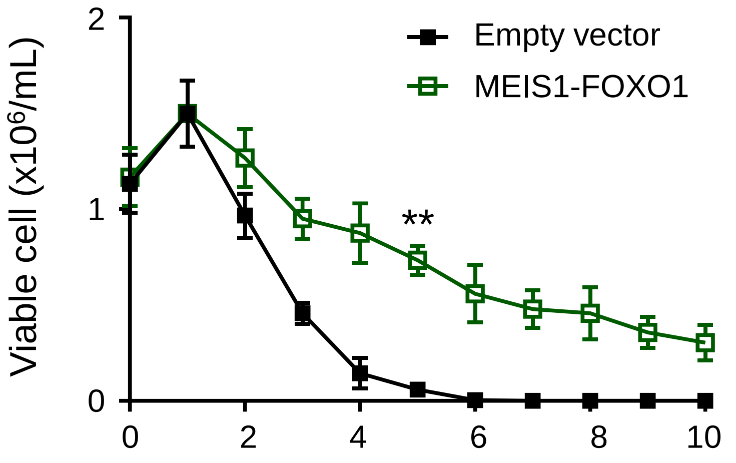

Supplementary Figure 3. MEIS1-FOXO1 fusion potentiated Ba/F3 cells survive as compared to Ba/F3 cells transduced with mock vector.

Supplementary Figure 4

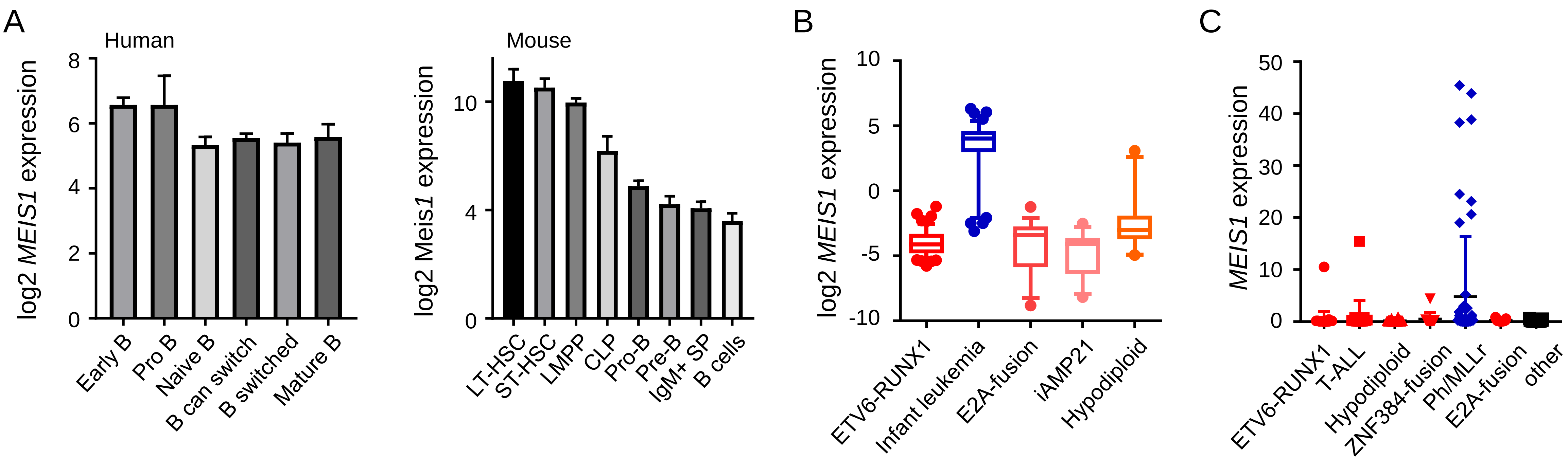

Supplementary Figure 4. *MEIS1* in normal hematopoiesis and acute lymphoblastic leukemia. (**A**) The MEIS1 transcription during human (left panel) and mouse (right panel) mouse B cells development. (**B-C**) The MEIS1 was merely expressed in childhood B-ALL.

Supplementary Figure 5

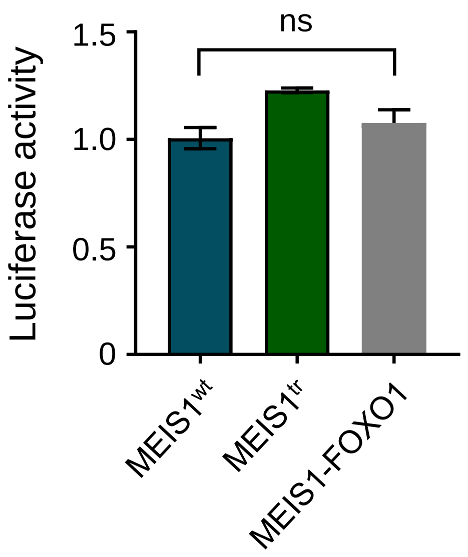

Supplementary Figure 5. The MEIS1 transcription activity of MEIS1-FOXO1 protein was not impaired.

Supplementary Figure 6

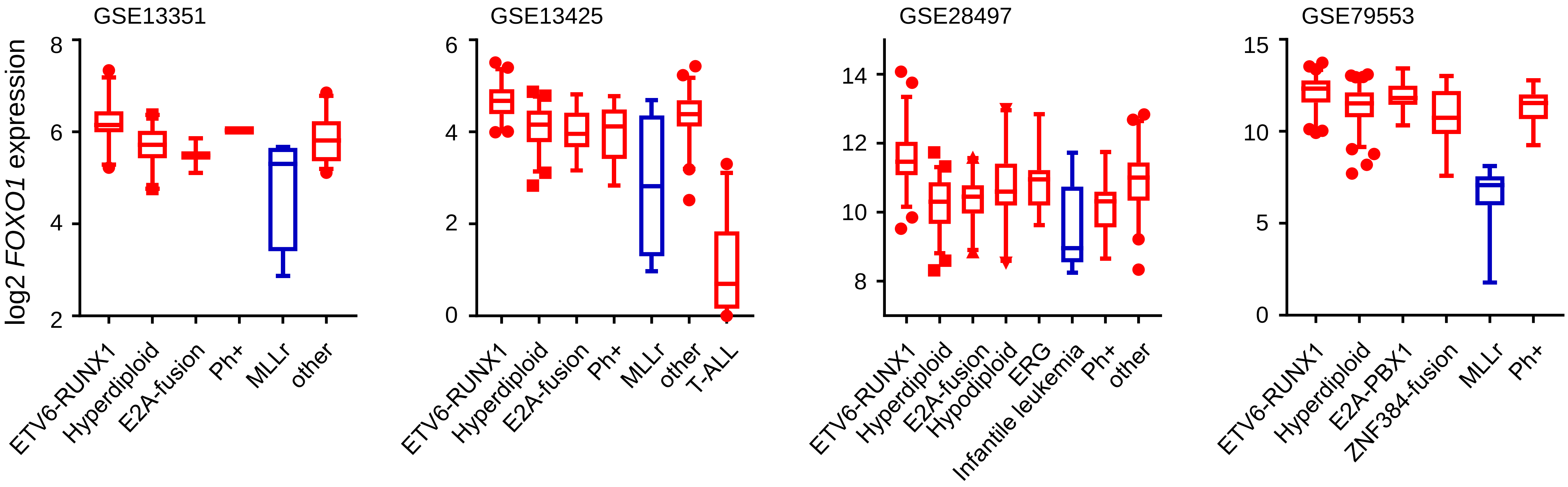

Supplementary Figure 6. *FOXO1* expression within different childhood ALL subtype.

Supplementary Figure 7

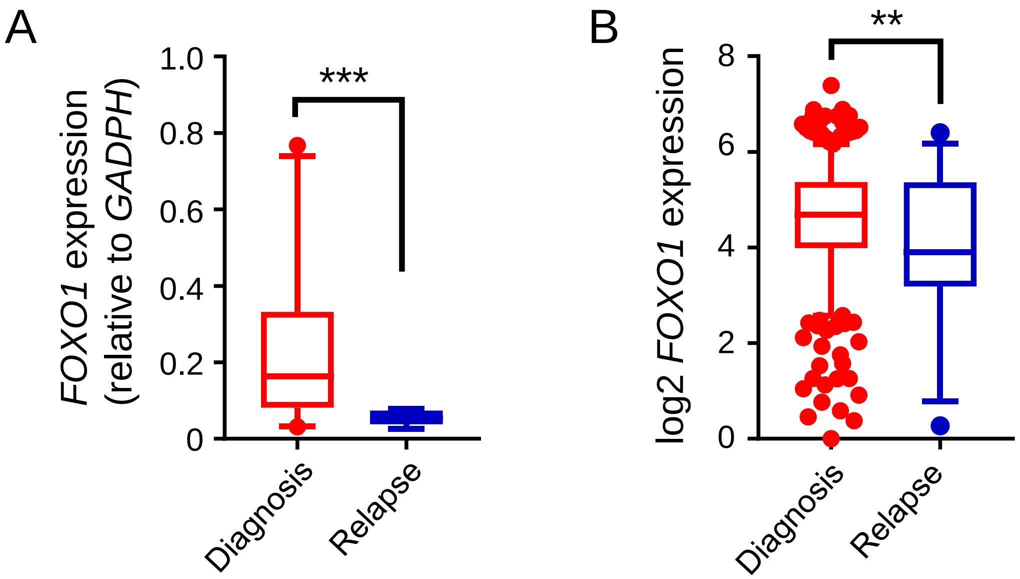

Supplementary Figure 7. *FOXO1* expression was lower in relapsed B-ALL than primary B-ALL samples.

Supplementary Figure 8

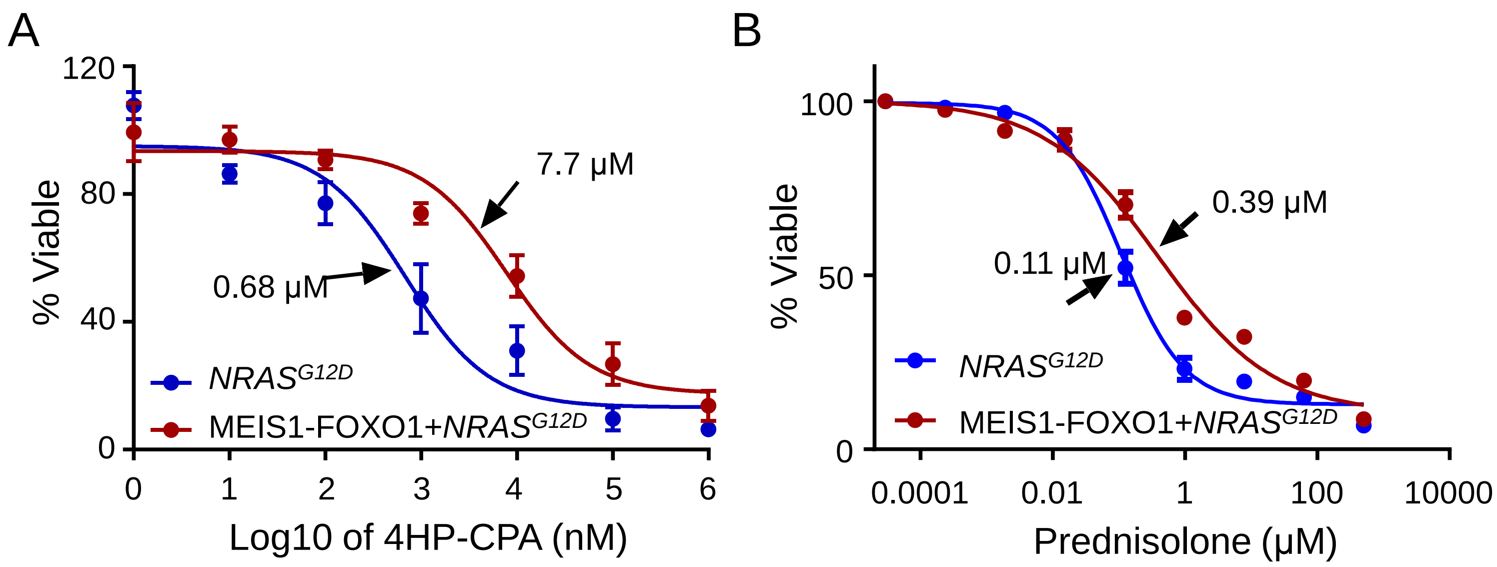

Supplementary Figure 8. Cytotoxicity of prednisone and 4HP-CPA was examined in Ba/F3 cells with *NRAS*^{G12D} expression [blue line] or combination of *MEIS1-FOXO1* and *NRAS*^{G12D} [dark red line].
